# New Insights into the Toxicokinetics of 3,4-Dichloroaniline in Early Life Stages of Zebrafish (*Danio rerio*)

**DOI:** 10.3390/toxics8010016

**Published:** 2020-03-01

**Authors:** Sabrina Schiwy, Ann-Kathrin Herber, Henner Hollert, Markus Brinkmann

**Affiliations:** 1 Department of Ecosystem Analysis, Institute for Environmental Research, ABBt–Aachen Biology and Biotechnology, RWTH Aachen University, 52074 Aachen, Germany; s.schiwy@bio5.rwth-aachen.de (S.S.); ann-kathrin.herber@rwth-aachen.de (A.-K.H.); henner.hollert@bio5.rwth-aachen.de (H.H.); 2 Department Evolutionary Ecology and Environmental Toxicology, Faculty Biological Sciences, Goethe University Frankfurt, 60438 Frankfurt, Germany; 3 State Key Laboratory of Pollution Control and Resource Reuse, School of the Environment, Nanjing University, Nanjing 210023, China; 4 College of Resources and Environmental Science, Chongqing University, Chongqing 400044, China; 5 Key Laboratory of Yangtze Water Environment, Ministry of Education, Tongji University, Shanghai 200092, China; 6 School of Environment and Sustainability, University of Saskatchewan, Saskatoon, SK S7N 5C8, Canada; 7 Toxicology Centre, University of Saskatchewan, Saskatoon, SK S7N 5B3, Canada; 8 Global Institute for Water Security, University of Saskatchewan, Saskatoon, SK S7N 3H5, Canada

**Keywords:** 3,4-DCA, biotransformation, FET, elimination rate constant

## Abstract

In the fish embryo toxicity (FET) test with zebrafish (*Danio rerio*) embryos, 3,4-dichloroaniline (3,4-DCA) is often employed as a positive control substance. Previous studies have characterized bioconcentration and transformation of 3,4-DCA in this test under flow-through conditions. However, the dynamic changes of chemical concentrations in exposure media and embryos were not studied systematically under the commonly used semi-static exposure conditions in multiwell plates. To overcome these limitations, we conducted semi-static exposures experiments where embryolarval zebrafish were exposed to 0.5, 2.0, and 4.0 mg L^−1^ of 3,4-DCA for up to 120 hpf, with 24-h renewal intervals. During each renewal interval, concentrations of 3,4-DCA were quantified in water samples at 0, 6, 18, and 24 h using high-performance liquid chromatography with diode array detection. Levels of 3,4-DCA in larvae were measured after 120 h exposure. Concentrations of 3,4-DCA in the test vessels decreased rapidly during exposure. Taking these dynamics into account, bioconcentration factors in the present study ranged from 12.9 to 29.8 L kg^−1^, depending on exposure concentration. In summary, this study contributed to our knowledge of chemical dynamics in the FET test with embryolarval zebrafish, which will aid in defining suitable exposure conditions for future studies.

## 1. Introduction

Chemical legislations around the globe mandate the risk assessment of chemicals to aquatic organisms [1,2,3]. These assessments typically make use of surrogate model species for three trophic levels: primary producers (algae), primary consumers (invertebrates), and higher-level consumers (fish) [4]. Intending to reduce the number of experiments with adult fish, recent decades have seen a significant movement towards trying to replace these experiments with early-life stage (ELS) models, which in many legislations are not considered animal experiments [5]. One such ELS model is the fish embryo toxicity (FET) test using embryolarval zebrafish. It has become a popular model for testing the toxicity of single chemicals, defined mixtures, and complex environmental samples [6]. Toxicity metrics obtained from the FET test show a strong positive correlation with those obtained through the acute fish test, indicating the acute fish test could potentially be replaced with the FET test. Thus, the FET test has also gained acceptance in the regulatory context. An Organisation for Economic Co-operation and Development (OECD) guideline was adopted, and an international multi-laboratory ring trial has demonstrated excellent performance across various laboratories [7]. However, the positive control that has traditionally been used in the test, 3,4-dichloroaniline (3,4-DCA), showed considerable variation in mortalities in the hands of these laboratories. Additionally, chemical analyses of real concentration in these experiments was performed in the absence of embryolarval zebrafish, but the concentrations of 3,4-DCA could have been affected by their presence (i.e., through biotransformation processes).

Thus, the present study aimed to investigate the underlying reasons for this highly variable behavior using a toxicokinetic approach. To this end, we conducted semi-static exposures experiments during which embryolarval zebrafish were exposed to three concentrations of 3,4-DCA (0.5, 2.0, and 4.0 mg L^−1^) for up to 120 h, with 24-h renewal intervals. During each renewal interval, levels of 3,4-DCA were quantified in water samples of four different time-points (0, 6, 18, and 24 h) using high-performance liquid chromatography with diode array detection (HPLC-DAD). Concentrations of 3,4-DCA in fish larvae were measured after 120 h exposure.

## 2. Materials and Methods

### 2.1. Chemicals and Stock Solutions

Unless stated otherwise, all chemicals (HPLC-grade) were obtained from Sigma-Aldrich (Steinheim, Germany). A stock solution of 3,4-DCA (100 mg L^−1^) was prepared in water and stored at 4 °C before using the preparation in the FET test.

### 2.2. Fish Maintenance and Egg Production

Zebrafish husbandry followed the procedures outlined by Braunbeck et al. [8], with modifications by Peddinghaus et al. [9]. The experiments were performed with the permission of federal and local authorities (Landesamt für Natur, Umwelt und Verbraucherschutz NRW, Germany; Amt für Verbraucherschutz, Tierschutz und Veterinärwesen, Städteregion Aachen, Würselen, Germany) and followed the Animal Welfare Act. Moreover, according to the EU Directive 2010/63/EU on the protection of animals used for scientific purposes, early-life stages of zebrafish are not protected as animals until the stage of being capable of independent feeding (<120 hpf) [10] [8]. For the benefit of our readers, however, we chose to refer to 120 hpf for the description of our last sampling time point.

Fish were maintained in glass aquaria using purified facility water (26 ± 1 °C, pH 7.8, hardness 11 dH) at a constant light: dark cycle of 14/10 h. The fish were fed ad libitum with dry flake food (TetraMin™; Tetra, Melle, Germany) and live Artemia sp. nauplius larvae (Silver Star Artemia, Inter Ryba GmbH, Zeven, Germany). A total of 20 three-month-old zebrafish were allocated to breeding groups at a sex ratio of 3:2 (males: females). Spawning trays were added to the tanks and consisted of a flat glass container with a metal mesh cover that was enriched with artificial plants. Mating and spawning occurred within 30 min after the light was switched on in the morning (Westerfield 2007). Embryos obtained from 5–10 tanks were pooled for each replicate.

### 2.3. Prolonged Fish Embryo Toxicity Test

The subsequent bioassays followed the recommendations of OECD Guideline 236, with modifications described by Schiwy et al. [11,12]. Briefly, fertilized fish eggs were visually selected using a binocular microscope (SMZ 1500, Nikon GmbH, Düsseldorf, Germany). Only normally developed fish embryos, at least in the 8-cell stage, were chosen for further testing. Normally developed embryos exhibited the following structure: The chorion surrounded the perivitelline space, which contained the yolk. The blastodisc was located at the animal pole of the yolk.

A total of 10 embryos were exposed to graded concentrations of 3,4-DCA (0.5, 2.0, and 4.0 mg·L^−1^) in 24-well microplates in two independent replicates, placing one embryo in 2 mL of medium (artificial water or different 3,4-DCA concentrations) per well. As negative control, embryolarval zebrafish were kept in artificial water (ISO 1996) only. Embryolarval zebrafish were exposed for up to 120 hpf at 26 ± 1 °C and microscopically inspected every 24 h using an inverted microscope at 40-fold magnification (Eclipse TS100, Nikon GmbH, Düsseldorf, Germany). Lethal endpoints (coagulation of the embryos/larvae, non-detachment of the tail, no heartbeat, and lack of somites) were recorded. Exposure solutions were exchanged every 24 h with fresh solutions. Exposure solutions from each well were sampled at four different time-points, pooled for each concentration level, and stored at −20 °C for subsequent chemical analysis. A “dummy plate” with triplicate wells of exposure solutions in the absence of embryolarval zebrafish was included to assess losses due to abiotic processes. Terminally, the hatched larvae were euthanized using benzocaine and stored at −20 °C for subsequent analysis of internal 3,4-DCA concentrations.

Tests were evaluated according to OECD Guideline 236 criteria. The FET was considered valid if the negative control did not show more than 10% mortality.

### 2.4. Determination of Aqueous and Internal 3,4-DCA Concentrations Using LC-DAD

Water samples were analyzed for the level of 3,4-DCA using a method previously published for the determination of NSO-heterocyclic compounds by Mundt & Hollender [13], with slight modifications. Liquid chromatography was performed using a 1200 Series LC chromatograph (Agilent, Waldbronn, Germany) equipped with a UV-diode array detector at 1.0 mL min^−1^ flow. Briefly, 40 µL subsamples were injected and separated on a Nucleosil C18 column at 40 °C (250 mm × 4 mm, 5 µm particle size; Macherey-Nagel, Düren, Germany). Gradient elution with acetonitrile (solvent A) and 5 mM potassium phosphate buffer at pH 7 (solvent B) was used to elute the chemical of interest. The following gradient program was used: 2-min hold at 10/90% (*v*/*v*), a 2-min ramp-up to 50/50% (*v*/*v*), an 8-min ramp-up to 60/40% (*v*/*v*), a 10-min ramp-up to 75/25% (*v*/*v*), up to 100/0% (*v*/*v*) in 5 min, back to 10/90% (*v*/*v*) in 12 min, followed by a 6-min hold for equilibration. Exposure water was thawed, filtered through a 0.45 µm glass fiber filter (Macherey-Nagel), and injected directly. Zebrafish larvae were thawed, homogenized in 1 mL ice-cold acetonitrile using an automatic disperser (VWR, Darmstadt, Germany), centrifuged (20 min, 4 °C, 4000× *g*; Hettich, Tuttlingen, Germany), and the supernatant injected into the HPLC system. Diode array detector signals at 210 and 254 nm, as well as the spectra from 190 to 400 nm, were recorded. The signal of 3,4-DCA was quantified at 254 nm. A four-point external calibration performed with 3,4-DCA (0.0, 0.5, 2.0, and 4.0 mg L^−1^) was used for interpolation of measured concentrations. These are the same concentrations as those used in the experiments with embryolarval zebrafish. Since water did not need to be extracted before analysis and was injected directly, it was not necessary to include internal standards.

### 2.5. Data Analysis

All raw spreadsheet data were analyzed in Microsoft Excel. Graphs were plotted and statistically analyzed using GraphPad Prism 8.3.1 software (GraphPad, San Diego, CA, USA, 2020). Measured 3,4-DCA concentrations from exposure experiments were analyzed using non-linear regression and fitted to a one-phase exponential decay model. Fitted dissipation rates (h^−1^ individual^−1^) and their standard error were calculated based on these fits. Furthermore, the areas under the curve were determined and used to calculate the time-weighted average (TWA) concentrations over the entire exposure period. Based on nominal and TWA concentrations, the bioconcentration factor (BCF) was calculated for each exposure concentration.

## 3. Results and Discussion

### 3.1. Survival of Zebrafish Larvae Exposed to 3,4-DCA

**Figure 1:**
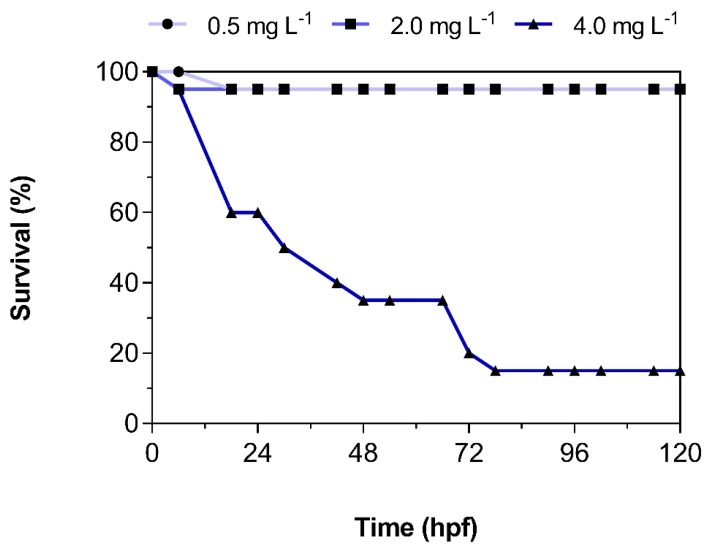
Percent survival of zebrafish embryolarval life stages exposed to 0.5, 2.0, and 4.0 mg L^−1^ 3,4-dichloroaniline (3,4-DCA). Data represent the average of *n* = 2 independent replicates.

The survival of embryolarval zebrafish was expected to be reduced following exposure to 3,4-DCA. At both nominal concentrations 0.5 and 2.0 mg L^−1^, no significant mortality (> 10%) was observed. With a value of 95%, survival remained high during the entire exposure period. At a nominal concentration of 4.0 mg L^−1^, survival decreased rapidly during the first 48 h and then plateaued at 35%, followed by a second decrease in survival down to 15% after hatch (Figure 1). These findings are in line with results from an international ring trial, which has found an average LC50 of 3,4-DCA of 3.2 mg L^−1^ at 48 h and 2.7 mg L^−1^ at 96 h, respectively [14]. As a result, the OECD Technical Guideline 236 recommends a concentration of 4.0 mg L^−1^ of 3,4-DCA as the positive control [11]. Similar two-phase decreases in survival following exposure to various chemicals (e.g., silica nanoparticles, single-walled carbon nanotubes) and sediment samples have been previously described in zebrafish [15,16,17]. However, we believe that this is the first time this behavior has been demonstrated for 3,4-DCA.

### 3.2. Dissipation of 3,4-DCA from Exposure Solutions

**Figure 2:**
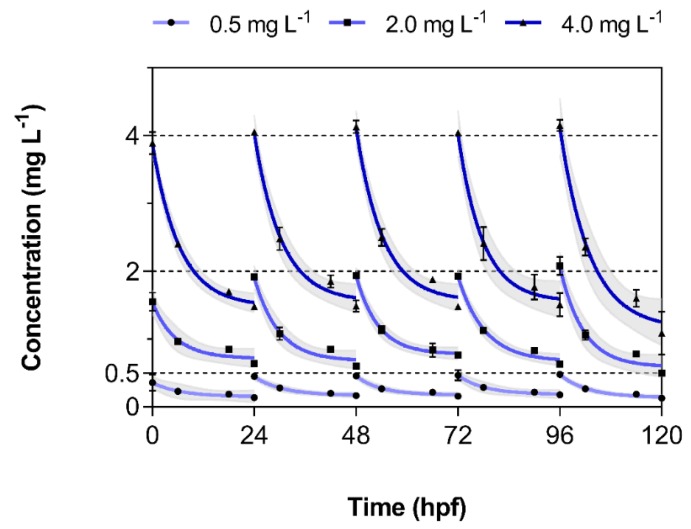
Measured concentration of 3,4-dichloroaniline (3,4-DCA) in exposure solutions during incubation with three different exposure concentrations (0.5, 2.0, and 4.0 mg L^−1^). Data represent the average ± standard deviation of measured 3,4-DCA concentration in *n* = 2 independent replicates. Dashed lines represent nominal exposure concentrations. The shaded area indicates the 95% confidence interval of the fitted one-phase exponential decay regression line.

Dissipation of 3,4-DCA from test vessels was observed during each of the five 24-h periods between water changes in intervals of 0, 6, 18, and 24 h, respectively (Figure 2). The data points obtained in this way were fitted using non-linear regression and generally followed first-order kinetics. Dissipation generally seemed to accelerate over time. Additionally, 3,4-DCA did not dissipate to a significant extent from the vessels in the absence of embryolarval zebrafish. The total dissipative losses in the absence of embryloarval zebrafish during a 24-h incubation period were 8.11 ± 1.94%, 13.4 ± 3.35%, and 4.89 ± 0.99% at 0.5, 2.0, and 4.0 mg L^−1^, respectively. The same trend was observed in the international ring trial [7]. These results might indicate that dissipation from test vessels was the result of biotransformation in embryolarval zebrafish.

**Figure 3:**
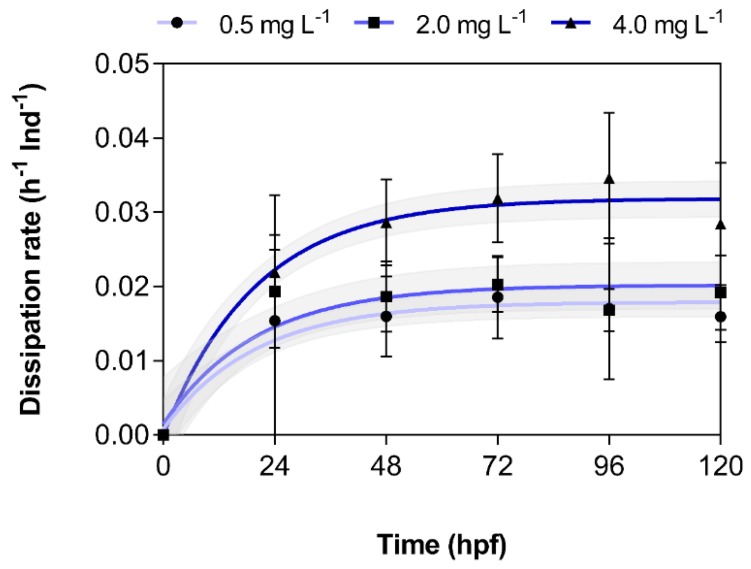
Dependence of the dissipation rates of 3,4-dichloroaniline (3,4-DCA) on exposure concentration and time. Rates were calculated based on the fitted mean and standard error of the one-phase exponential decay data presented in Figure 2 (*n* = 2 independent replicates). Shaded area indicates the 95% confidence interval of the regression lines.

Thus, based on the results of the regression analysis of dissipation data for each of the five 24-h intervals, the mean and standard error for the interpolated first-order rate constants were determined and plotted over time (Table 1, Figure 3). Indeed, there was a marked increase in dissipation rates over time and with increasing exposure concentration, which further suggests that biotransformation might be the cause of this dissipation. Similarly, high dissipation rates were not observed in the absence of embryolarval zebrafish (data not shown) and have also not been described during the OECD ring trial [7].

### 3.3. Bioconcentration of 3,4-DCA in Exposed Zebrafish Larvae

**Figure 4:**
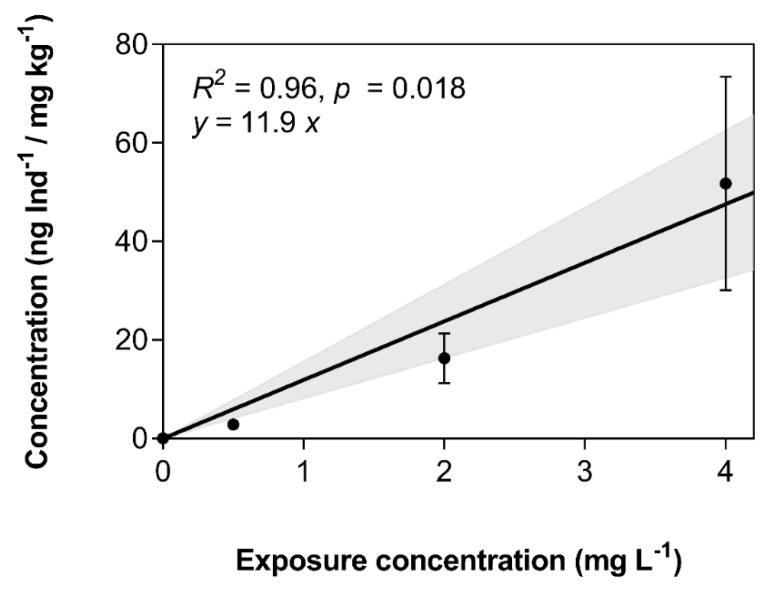
Linearity of bioconcentration of 3,4-dichloroaniline (3,4-DCA) depending on exposure concentration. Data represent the average ± standard deviation of the 3,4-DCA concentration in all surviving larvae after 120 hpf of *n* = 2 independent experiments. The regression equation, as well as Pearson’s correlation metrics, are provided. The shaded area indicates the 95% confidence interval of the regression line. Larvae of zebrafish 120 hpf weighed 1.0 mg wet weight; in consequence, concentrations on the y-axis translate directly to mg kg^−1^.

Internal concentrations in exposed zebrafish larvae were determined after 120 hpf. These increased in a concentration-dependent manner from 2.83 ± 0.57 to 16.3 ± 5.12 to 51.7 ± 21.7 ng Ind^−1^ larva at 0.5, 2.0, and 4.0 mg L^−1^, respectively (Table 1, Figure 4). Larvae of zebrafish at 120 hpf had a wet weight of 1.0 mg. In consequence, concentrations in ng Ind^-1^ translate directly to ng mg^−1^ or mg kg^−1^. Based on dissipation data from Figure 2, the time-weighted average (TWA) concentrations were determined for each exposure concentration (Table 1). TWA concentrations were generally about two-fold lower compared to nominal concentrations. Bioconcentration factors (BCFs) were calculated based on nominal and TWA concentrations (Table 1). Because of the lower corresponding aqueous concentrations used in the calculation, TWA-based BCFs were approximately two-fold higher compared to those based on nominal concentrations. Furthermore, BCFs increased with exposure concentration, which might indicate that the biotransformation enzymes of embryolarval zebrafish are increasingly saturated at these high exposure concentrations (Table 1). Generally, BCFs measured in the present study were two- to three-fold lower compared to a value of 86 previously published by Hertl & Nagel [18]. However, given that the present study was conducted at considerably higher concentrations compared to the approximately 16 to 80 µg L^−1^ in the Hertl & Nagel study [18], and did not strictly adhere to the respective OECD guideline, this discrepancy is not surprising [19]. It is recommended that BCF studies be conducted at concentrations as low as possible [19]. However, accurate BCF estimation was not the primary goal of the present study, and the reported BCFs should not be used as such in a regulatory context.

### 3.4. Conclusions and Relevance for the FET Test

In summary, it could be shown that the aqueous exposure concentration of 3,4-DCA in the FET test decreased rapidly during the 24-h intervals between changes of exposure solutions. We present evidence that suggests that this rapid decrease was, at least in part, due to biotransformation in embryolarval zebrafish. Thus, we recommend that exposure concentration should not only be verified in exposure vessels in the absence of embryos/larvae, but rather in the presence of embryos/larvae as well. This way, abiotic and biotic dissipative and transformative processes can be captured reliably. These measurements should, in the best case, include several sampling points between each change of exposure solutions.

## Figures and Tables

**Table 1:** Nominal exposure concentrations, time-weighted average (TWA) exposure concentrations based on the area-under-the-curve (AUC) approach, measured internal concentrations in larvae 120 hpf (as larvae of that age weighed 1 mg wet weight, levels in ng Ind^−1^ translate directly into mg kg^−1^), as well as bioconcentration factors based on nominal and TWA concentrations, and dissipation rates from exposure vessels (*n* = 2 independent replicates).

Nominal Concentration (mg L^−1^)	Time-Weighted Average Concentration (mg L^−1^)	Internal Concentration (ng Ind^−1^/ mg kg^−1^)	Bioconcentration Factor (Nominal) (L kg)	Bioconcentration Factor (TWA) (L kg)	Maximum Dissipation Rate (h^−1^ Ind^−1^)
0.50	0.25	2.83 ± 0.57	5.66 ± 1.13	11.3 ± 2.27	0.018 ± 0.001
2.00	1.03	16.3 ± 5.12	8.13 ± 2.56	15.8 ± 4.97	0.020 ± 0.002
4.00	2.25	51.7 ± 21.7	12.9 ± 5.42	23.0 ± 9.64	0.032 ± 0.001
